# Diverse Transcriptome Responses to Salinity Change in Atlantic Cod Subpopulations

**DOI:** 10.3390/cells12232760

**Published:** 2023-12-03

**Authors:** Magdalena Małachowicz, Aleksei Krasnov, Roman Wenne

**Affiliations:** 1 Institute of Oceanology Polish Academy of Sciences, Powstanców Warszawy 55, 81-712 Sopot, Poland; mwarzecha@iopan.pl; 2 Department of Fish Health, Nofima—Norwegian Institute of Food, Fisheries and Aquaculture Research, Osloveien 1, NO-1431 Ås, Norway; aleksei.krasnov@nofima.no

**Keywords:** *Gadus morhua*, osmoregulation, gene expression, microarray, gill tissue

## Abstract

Adaptation to environmental variation caused by global climate change is a significant aspect of fisheries management and ecology. A reduction in ocean salinity is visible in near-shore areas, especially in the Baltic Sea, where it is affecting the Atlantic cod population. Cod is one of the most significant teleost species, with high ecological and economical value worldwide. The population of cod in the Baltic Sea has been traditionally divided into two subpopulations (western and eastern) existing in higher- and lower-salinity waters, respectively. In recent decades, both Baltic cod subpopulations have declined massively. One of the reasons for the poor condition of cod in the Baltic Sea is environmental factors, including salinity. Thus, in this study, an oligonucleotide microarray was applied to explore differences between Baltic cod subpopulations in response to salinity fluctuations. For this purpose, an exposure experiment was conducted consisting of salinity elevation and reduction, and gene expression was measured in gill tissue. We found 400 differentially expressed genes (DEGs) involved in the immune response, metabolism, programmed cell death, cytoskeleton, and extracellular matrix that showed a subpopulation-dependent pattern. These findings indicate that osmoregulation in Baltic cod is a complex process, and that western and eastern Baltic cod subpopulations respond differently to salinity changes.

## 1. Introduction

Global climate change due to human activities has an impact on many environmental parameters, including salinity. Salinity fluctuation is a significant climate change stressor affecting the abundance, diversity, distribution, growth, osmolarity, and metabolic activities of fish and other marine organisms [1]. Seawater salinity has been decreasing in some coastal seas due to the increased intensity of rains, freshwater inflow, and ice melting [2]. One of the regions most threatened by global climate change is the Baltic Sea, where deviation in atmospheric circulation in the early 1980s reduced the inflow of Atlantic oxygenated, cold, and salty water, thus causing dramatic changes in the ecosystem in the following decades [3]. According to physical–biogeochemical models, due to the increasing precipitation over Scandinavian land masses and the limited Atlantic Ocean connection, salinity in the Baltic Sea will continue to decrease in the coming decades [4,5,6]. For these reasons, the Baltic Sea has been used as a world-renowned model for environmental changes [7]. The hazards mentioned above result from, among others, the characteristics of these waters. The Baltic Sea is a shallow, semi-enclosed, brackish sea characterized by gradually decreasing salinity in the northeast direction from 35 ppt in the Atlantic Ocean to about 8 ppt in the southern Baltic Sea, and to 2–3 ppt in the inner Gulf of Bothnia, with considerable local short-term salinity variations [5,8,9]. It is also characterized by high vertical salinity stratification; the deep water mass has a higher salinity than at the surface and it is less oxygenated. Due to such a challenging environment, only a few marine fish species have adapted to these conditions, including Atlantic cod (*Gadus morhua* L.).

The Atlantic cod is a key euryhaline fish species in the North Atlantic Ocean, with significant commercial and ecological value that will be threatened by the expected climate-related decline in salinity in the Baltic Sea [10,11]. Cod adapt to a wide range of salinities, from 35 ppt in the North Atlantic, 20 ppt in the surface waters of Kattegat and the western Baltic, to about 3 ppt in the northeastern Baltic; thus, they provide an excellent model to study the molecular mechanisms underlying salinity adaptations. Previous studies using single-nucleotide polymorphisms (SNPs), microsatellites, and transcriptomic data revealed a genetic distinctiveness between the Baltic and Atlantic cod populations [12,13,14]. Further, several molecular studies [15,16,17,18,19] confirmed the existence of two separate subpopulations in the Baltic Sea: one termed western Baltic cod (abbreviated as WBC), located in subdivisions (SDs) 22–24, and the other termed eastern Baltic cod (EBC), located in SDs 24–32 (division by the International Council for the Exploration of the Sea (ICES)). Due to these results, the inner Baltic cod is monitored and managed as western and eastern stocks for fishery purposes [19,20].

Adult Baltic cod often undertake complex migrations to feeding and spawning grounds depending on the area [21]. According to previous studies, Baltic cod require a salinity above 11–12‰ for egg development and successful reproduction [22], which limits spawning areas to several locations. The main spawning areas for WBC are Kiel Bay and Arkona Basin. The latter is also used by EBC, which can result in mixing [20]. Former spawning grounds for the eastern stock in Gdańsk and Gotland Deep are dysfunctional because of strongly reduced inflows of high salinity and oxygenated water from the North Sea and increased hypoxia; thus, reproduction mainly takes in the Bornholm Basin [23,24,25]. During vertical and horizontal migrations to spawning areas with a depth occupation of over 60 m and active travelling speed of up to 14 m d^−1^, Baltic cod is exposed to variable salinities [26]. For successful spawning, EBC enter deep water up to 19.2 ppt, as revealed by tagging experiments [27]. Due to challenging brackish conditions in the Baltic Sea, cod subpopulations exhibit several physiological and genetic adaptations to low-salinity water, including egg buoyancy [28], sperm motility [29], hemoglobin type [30], osmoregulation and ion exchange [31], and different spawning times [21]. Studies on the Atlantic cod response to salinity stress have been focused on selected osmoregulatory genes such as Na/K-ATPase α genes (*atp1a*) and heat shock protein 70 (*hsp70*), aquaporins, the solute carrier gene family, and receptors of prolactin (*prl*) [16,31,32]. Further, recent studies showed that the genes involved in adaptation to low salinity in the Baltic Sea are located on chromosomes 2 and 12 [16,18,33]. These adaptations to the environmental conditions in the Baltic Sea may contribute to a strong and effective reproductive barrier between WBC and EBC; thus, Baltic cod can be viewed as an example of ongoing speciation [16]. Our preliminary studies of the transcriptome showed that eastern and western Baltic subpopulations from the natural environment differ in gene expression, which might be related to salinity tolerance [13,34]. However, understandings of the local genetic adaptation to low salinity in the Baltic Sea are still limited. Further, in recent decades, the Baltic cod population has decreased, especially EBC, which have experienced a strong depletion manifested by decreased resistance to pathogens, length–weight factor, reproduction/productivity, and an increase in natural mortality [25,35]. The reason for this, alongside overfishing, is the change in climate and environmental conditions, including salinity, which has caused a reduction in spawning areas.

Although many studies have investigated the response of the fish transcriptome to salinity changes using next-generation sequencing (NGS) [36,37,38] and microarrays [5,39,40], the current understanding of genetic and physiological responses to salinity fluctuations is still limited. In general, teleosts use similar strategies to maintain osmotic homeostasis; however, there are also some differences in the regulation of internal water and solute homeostasis [41]. Furthermore, recent studies revealed inter-population differences in fish responses to salinity [42,43]. To address this problem in the present study, cod individuals derived from the western and eastern subpopulations were kept in tanks, where salinity was gradually changed, and then gene expression was measured using a genome-wide DNA oligonucleotide microarray to characterize the putative salinity-regulated genes and the biological and molecular processes involved in salinity adaptation.

## 2. Materials and Methods

### 2.1. Ethics Approval

The experimental procedures were carried out according to the EC Directive 2010/63/EU for animal experiments and were approved by the Local Ethics Committee on Animal Experimentation of Gdansk Medical University (decision no. 60/2012).

### 2.2. Sampling and Experimental Design

**Figure 1:**
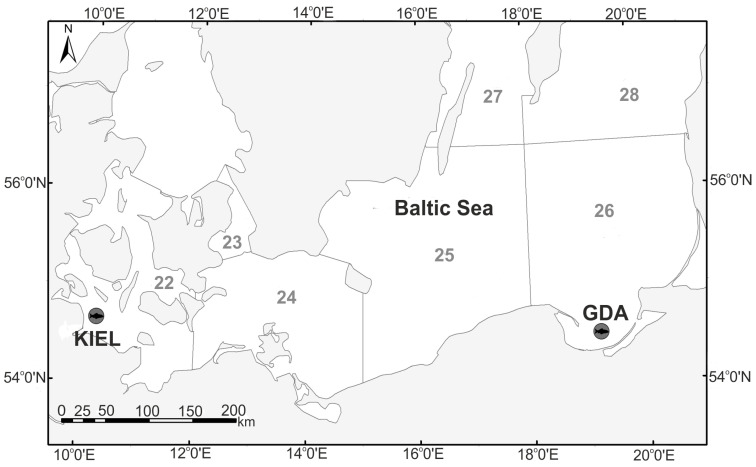
Sampling locations. The fish of the Kiel Bight (KIEL) and the Gulf of Gdańsk (GDA) represent the eastern and western Baltic cod subpopulations, respectively.

*Gadus morhua* juvenile individuals were caught in November 2012 in Schleifjord, Kiel Bight (KIEL)—ICES SD 22, with an average size of 33.95 ± 0.32 cm, and the Gulf of Gdańsk (GDA)—SD 26, with an average size of 30.7 ± 5.27 cm, which represent the western and eastern Baltic cod subpopulations, respectively (Figure 1).

**Figure 2:**
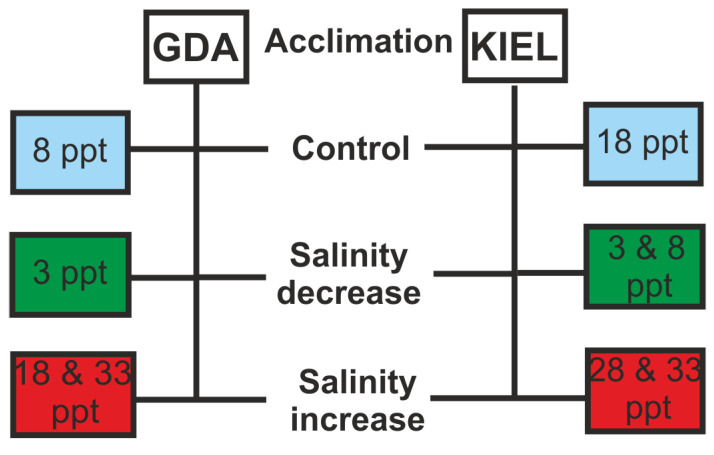
Experimental design using *Gadus morhua* individuals of two geographical origins: the Gulf of Gdańsk (GDA) and the Kiel Bight (KIEL), representing eastern and western Baltic cod, respectively.

The live fish were transported in containers with sea water to the marine station of the University of Gdańsk in Hel, and then divided into two 2000 L tanks filled with recirculated water. The fish were kept at a constant temperature (10 °C), in a natural photoperiod, and under salinity conditions similar to those of the collection sites (GDA—8 ppt; KIEL—18 ppt). They were fed fresh herrings once a day and acclimated for more than 14 days until their typical behavior was restored. Next, fish from each localization were randomly divided and transferred to separate tanks termed control, salinity decrease, and salinity increase groups, and again acclimated as described above (Figure 2). The control tanks represented the natural salinities of the geographic region (abbreviated as GDA8 and KIEL18). During the osmotic challenge, the salinity was gradually changed (1 ppt per hour) in order to exclude acute stress and was measured every hour with a conductometer (Elmetron, Zabrze, Poland) [32]. In the increase group, salinity was elevated by 10 ppt and to 33 ppt in the GDA and KIEL groups (abbreviated as GDA18, GDA33, KIEL28, and KIEL33; Figure 2) by adding aquarium ocean salt (Aquarium Systems, Sarrebourg, France). In other tanks, salinity decreased by 10 ppt in the KIEL group (KIEL8) and to 3 ppt in both the GDA and KIEL groups (GDA3 and KIEL3). In experimental tanks, individuals were collected after 1 h, 12 h, and 72 h after reaching the desired salinity level (Figure 2). The fish were anesthetized using tricaine methanesulfonate (MS222) and sacrificed via spinal cord dissection, and their weights and lengths were measured.

### 2.3. RNA Extraction

Samples of gill were collected from 81 fish: KIEL (n = 48) and GDA (n = 33) and stored in RNAlater, according to the manufacturer’s instructions (Qiagen, Hilden, Germany) [34]. Total RNA was extracted with the ISOLATE II RNA Mini Kit (Bioline, London, UK) and stored at −80 °C [14]. The RNA concentration and integrity were measured with the Epoch microplate spectrophotometer (BioTek Instruments, Inc., Winooski, VT, USA) and Agilent Bioanalyser (Agilent, Santa Clara, CA, USA) [13]. The mean RNA integrity value was 9.62 ± SD 0.59 for the KIEL and 9.05 ± SD 0.93 for the GDA groups.

### 2.4. Microarray Analysis

Microarray hybridization was performed in the Department of Physiological Sciences of Warsaw University of Life Sciences (SGGW) with the Agilent-0048047 Genome-wide Atlantic cod microarray, second version_ACIQ2 (GEO accession no. GPL18775; Agilent, Santa Clara, CA, USA) using the Gene Expression Hybridization Kit (Agilent, Santa Clara, CA, USA), according to the manufacturer’s protocol. This DNA oligonucleotide 4 × 44 k microarray includes 60 mer probes for unique transcripts from the Ensembl and Unigene databases [44]. The hybridized arrays were washed using the Agilent Gene Expression Wash Buffer Kit and scanned with an Agilent Technologies Scanner G2505C, according to the manufacturer’s protocol (GE2_1010_Sep10). The scanned microarray images were analyzed using Agilent Feature Extraction software (version 10.10.1.1) [34]. Normalization of the raw mean signal was performed using the Limma package (version 3.58.1) in R [34,45] using the following functions: BackgroundCorrect (normexp method), normalizeWithinArrays (lowess method), and normalizeBetweenArrays (quantile method) [34]. Differentially expressed genes (DEGs) between the experimental groups and the control were identified using the significance analysis of microarrays (SAM) method in siggenes software (version 1.76.0) [46] and the rank product method in the RankProd package (version 3.28.0) [47], with a fold-change [FC] ≥ 2, a *p*-value < 0.05, and a false discovery rate (FDR) < 0.05. The Pearson correlation between experimental groups was analyzed using the R package. The Wilcoxon rank sum test (in R) was used to compare the means of the GDA and KIEL groups. Normalized data were analyzed using a two-factor model implemented in the limma package in R (*p*-value < 0.05) to assess the time course of responses to salinity changes in subpopulations (the factors were population and time or salinity).

Available gene annotations were downloaded from the Ensembl database (release 99; http://www.ensembl.org/Gadus_morhua, accessed on 1 February 2020). Genes without annotations were searched against proteins from the NCBI non-redundant (nr) database using the Basic Local Alignment Search Tool (blastx) implemented in BLAST+ (v.2.2.29) [48], with an E-value threshold of 10^−5^, using sequences to which probes were designed [34]. Gene symbols were assigned using the HUGO Gene Nomenclature Committee (HGNC) and the Zebrafish Information Network (ZFIN) databases. Pathway enrichment analysis was performed using the *Kyoto Encyclopedia of Genes and Genomes* (KEGG) Orthology-Based Annotation System (KOBAS v.3.0) for the human data (corrected *p*-value < 0.05) [49].

### 2.5. Data Availability

The microarray data were deposited in the National Center for Biotechnology Information Gene Expression Omnibus (NCBI GEO) under the accession number GEO: GSE195878.

## 3. Results

### 3.1. Measurements

**Figure 3:**
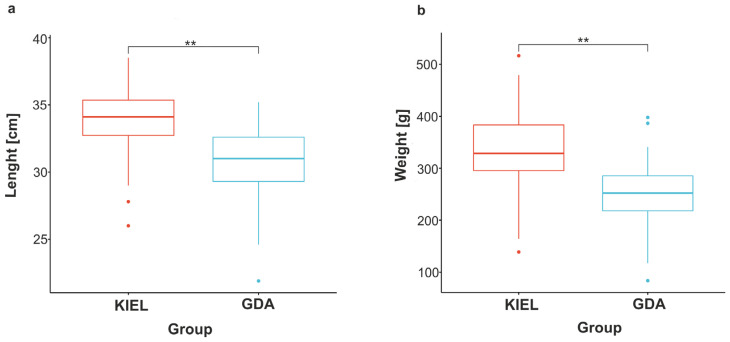
Measurements of the (**a**) length and (**b**) weight of the individuals used in this study (n = 81). The Gulf of Gdańsk (GDA) represents EBC; the Kiel Bight (KIEL) represents WBC. Two asterisks indicate a *p*-value < 0.001.

The length and weight of each fish used in this experiment were measured and compared between the GDA and KIEL groups using the Wilcoxon rank sum test. Statistical analysis revealed that the body parameters of the GDA fish were significantly lower than those of the KIEL group (*p*-value < 0.001; Figure 3).

### 3.2. Microarray Analysis

**Figure 4:**
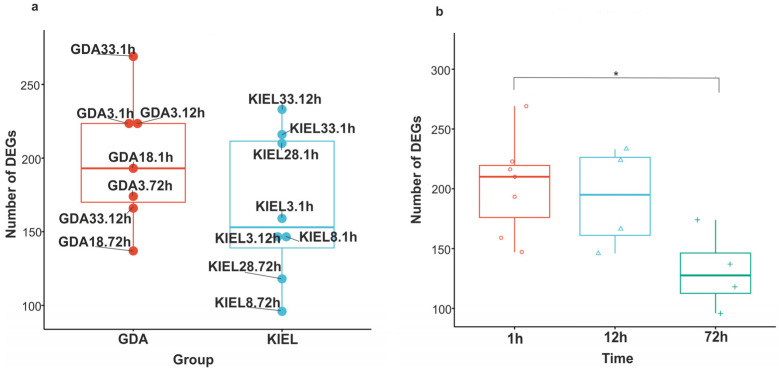
Number of DEGs in the each experimental group. The Gulf of Gdańsk (GDA) represents EBC; the Kiel Bight (KIEL) represents WBC. (**a**) Number of DEGs in each experimental group. (**b**) No. of DEGs in time course. An asterisk represents a *p*-value < 0.05.

Comparison with references from both localizations revealed differentially expressed genes in each experimental group (including time and salinity). In summary, 756 DEGs and 881 DEGs were identified in the GDA and KIEL subpopulations, respectively (Supplementary Table S1). Of these, on average, 66.86% were annotated using the Ensembl or NCBI nr databases (excluding uncharacterized and hypothetical proteins), and gene symbols were assigned to 58.05%. The analysis revealed that subpopulations shared 296 DEGs (Venn diagram, Supplementary Figure S1). The numbers of DEGs in each experimental group (including salinity and time) are presented in Figure 4 and Supplementary Table S2. In general, a lower number of DEGs was identified across the KIEL groups compared to the GDA groups; however, the difference was not statistically significant (Figure 4a). The increase to 33 ppt in KIEL (12 h) and GDA (1 h) caused the expression changes in the highest number of genes compared to other groups (Figure 4a). The number of DEGs was similar between 1 h and 12 h and then decreased after 72 h (1 h vs. 72 h; *p*-value < 0.05) in both the KIEL and GDA groups (Figure 4b).

**Figure 5:**
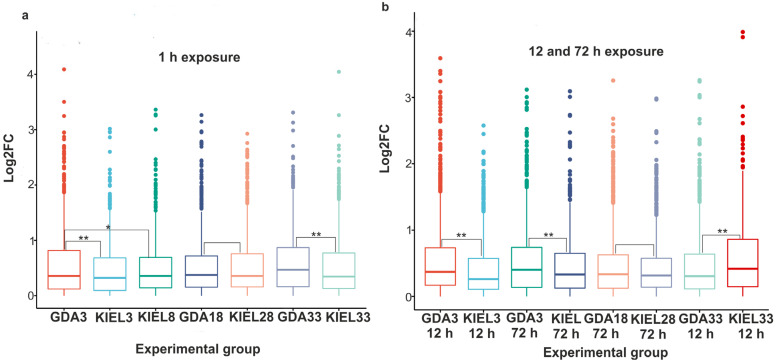
Differences in expression between the GDA and KIEL groups (Wilcoxon rank sum test). (**a**) Expression after 1 h of exposure to salinity change. (**b**) Expression after 12 and 72 h of exposure to the change in salinity. An asterisk represents a *p*-value < 0.05; two asterisks denotes a *p*-value < 0.001. The Gulf of Gdańsk (GDA) represents EBC; the Kiel Bight (KIEL) represents WBC.

A positive Pearson correlation was found between the experimental groups within the subpopulations, indicating a similar expression trend (Supplementary Figure S2a). The opposite results were obtained by comparing the GDA and KIEL groups, which showed a negative correlation (Supplementary Figure S2a). Wilcoxon’s rank sum test using the absolute value of log2 expression revealed that in most GDA and KIEL groups the median expression level was significantly higher at 1 h compared to 12 h or 72 h; however, in the KIEL8 (1 h vs. 72 h) and GDA3 (1 h vs. 12 h) groups, the difference was not significant. It was different in the KIEL33 group, where median expression increased with time (*p*-value < 0.05; Supplementary Figures S2b and S2c). Furthermore, the magnitude of the expression changes in the KIEL group after 1 h of exposure to increased salinity was higher (*p*-value < 0.001), following which salinity decreased (Supplementary Figure S2c). There was no such trend in the GDA groups. In the event that salinity decreased and increased to 33 ppt, the magnitude of expression changes after 1 h of exposure was higher in the GDA group (Figure 5a). The same pattern was revealed when comparing the groups with reduced salinity after 12 h and 72 h; however, a salinity level increase to 33 ppt provoked higher expression in the KIEL group compared to the GDA group (*p*-value < 0.001). Salinity increased by 10 ppt caused similar magnitude expression changes in both the GDA and KIEL groups at each time point (Figure 5a,b).

**Figure 6:**
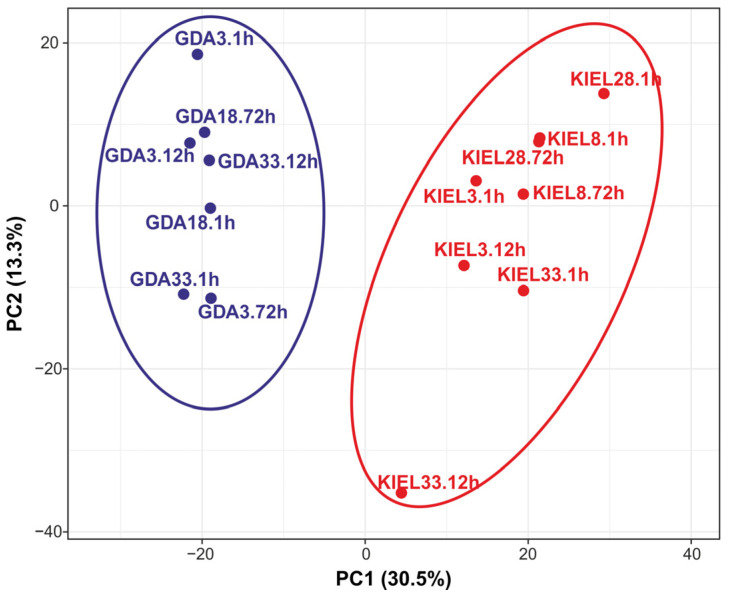
Principal component analysis (PCA) performed for all DEGs. Blue color: the Gulf of Gdańsk (GDA) represents EBC; red color: the Kiel Bight (KIEL) represents WBC.

The principal component analysis (PCA) performed for all identified DEGs from this study showed a clear division of the GDA and KIEL groups (Figure 6).

### 3.3. Sub-Population-Dependent Transcripts

**Figure 7:**
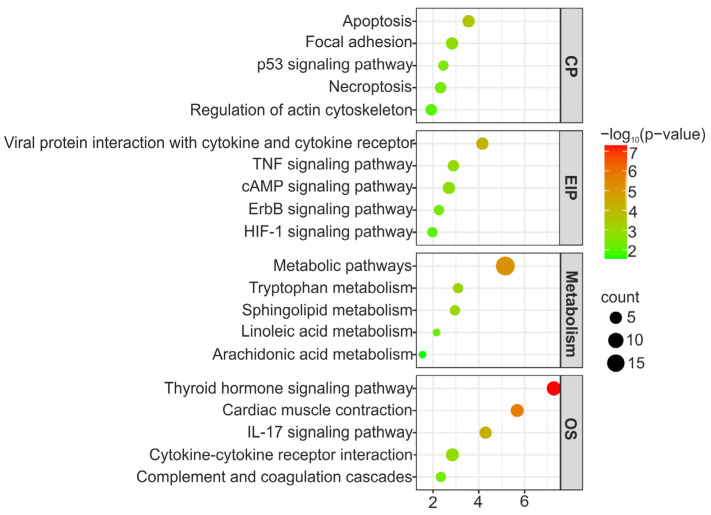
Bubble plot of pathway enrichment analysis using subpopulation-dependent genes. The plot presents the top five pathways, with the highest *p*-value divided into the main KEGG categories. CP: cellular processes; EIP: environmental information processing; and OS: organismal systems.

Across all transcripts included in the analysis, 29.83% (400) showed differential expression between subpopulations (FC > 2; Supplementary Table S3). Of these, 70.50% were annotated, and 57% had a gene symbol. The enrichment analysis of subpopulation-dependent genes revealed several DEGs involved in the immune response (complement and coagulation cascade, the ILl-17 signaling pathway, cytokine–cytokine receptor interaction, and neutrophil degranulation; Figure 7). These included *cd59*, complement factor H (*cfh*), chemokines (*ccl2*, *ccl20*, and *ccr2*), mucins (*muc2*, *muc5b*, and *muc5ac*), lectins toll-like receptor 2 (*tlr2*), nlr family card and pyrin domain containing 3 (*nlrc3* and *nlrp3*), and natterin (*aep1*). Endocrine system pathways, such as thyroid hormone signaling, PPAR signaling, and estrogen signaling, were also enriched across subpopulation-dependent genes. Between these genes, there were several Ca^2+^-transporting ATPases (*atp2a1* and *atp2a2*), protein kinase c alpha (*prkca*), phosphatidylinositol-4,5-bisphosphate 3-kinase catalytic subunit alpha (*pik3ca*), notch receptor 2 (*notch2*), iodothyronine deiodinase 1 (*dio1*), and creb-binding protein (*crebbp*). The EBC and WBC subpopulations also showed differential regulation of genes involved in lipid metabolism (sphingolipid metabolism, arachidonic acid pathways, and linoleic acid pathways), such as ceramides (*cers2*, *cers3*, and *cers4*), phospholipase a and acyltransferase 3 and 4 (*plaat3* and *plaat4*), phospholipase d family member 4 (*pld4*), prostaglandin i2 synthase (*ptgis*), farnesyl diphosphate synthase (*fdps*), fatty acid-binding protein 1 (*fabp1*), acyl-coa synthetase bubblegum family member 2 (*acsbg2*), and two genes of the cytochrome p450 family 1 subfamily a (*cyp1a1* and *cyp1a2*). Furthermore, genes involved in programmed cell death (apoptosis, necroptosis, and the p53 signaling pathway), including caspases (*casp2*, *casp8,* and *casp13*), bcl2-associated x, apoptotic regulators (*bax*), bcl2-like 14 (*bcl2l14*), bcl2-interacting protein 3 (*bnip3*), and members of the tnf receptor superfamily (*tnfrsf9*, *tnfrsf10b*, *tnfrsf14*, and *tnfrsf15*), were differentially expressed between these two subpopulations. The western and eastern subpopulations also showed differential regulation of genes related to tissue remodeling, such as type X collagen (*col10a1*), type 13 keratin (*krt13*), fibronectin 1 (*fn1*), lysyl oxidase (*lox*), tenascin n (*tnn*), myosin heavy chain 3 and 7 (*myh3* and *myh7*), and myosin light chain 3 and 10 (*myl3* and *myl10*), which are involved in the regulation of the actin cytoskeleton (Figure 7).

**Figure 8:**
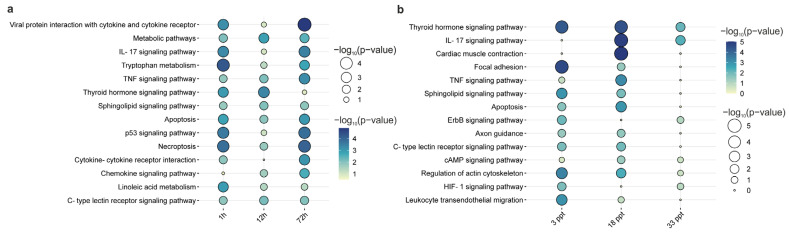
Changes in the enrichment of the top pathways of subpopulation-dependent genes. (**a**) Time course and (**b**) salinity change.

Of the total of 290 DEGs with time differences, 75 (25.86%) were shared between time points, including such transcripts as *bax*, *casp8*, kinesin family member 5b (*kif5b*), *nlrp3, pik3ca*, and *tnfrsf10b* involved in apoptosis and necroptosis, *cyp1a1*, *cyp1a2*, and *acsbg2* involved in fatty acid metabolism, and *dio1* and *notch2* related to the thyroid hormone signaling pathway. Additionally, several immune-related genes such as *aep1*, *ccl20*, and interferon-induced very large GTPase 1 (*gvinp1*), while *fish-egg lectin*, *muc2*, and *piscidin* were regulated over time (Figure 8a). A suite of immune genes such as *lysozyme g*, *muc5ac*, s100 calcium-binding protein *p* (*s100p*), sam and hd domain containing deoxynucleoside triphosphate triphosphohydrolase 1 (*samhd1*), *tnfrsf14*, and *tnfrsf15*, as well as the stress response protein (*hsp70*) and genes involved in glycerophospholipid biosynthesis (*plaat3* and *plaat4*) and haloacid dehalogenase-like hydrolase domain containing 5 (*hdhd5)* were differentially expressed only 1 h after the change in salinity. Further, 12 h after the salinity change, genes involved in neutrophil degranulation, such as rab37, members of the ras oncogene family (*rab37*), actin-related protein 2 (*actr2*), purine nucleoside phosphorylase (*pnp*), and *tlr2*, showed differences between the GDA and KIEL groups, as well as *mhc class I anigen*. Moreover, genes involved in the cell cycle, including the up-regulator of cell proliferation-like (*urgcp*) and nedd1 gamma-tubulin ring complex targeting factor (*nedd1*), and in tyrosine degradation, such as dihydropteridine reductase (*qdpr*) and asparaginase and isoaspartyl peptidase 1 (*asrgl1*), were only differentially expressed after 12 h. On the other hand, aldolase fructose-bisphosphate a (*aldoa*) (involved in glycolysis), *pld4* (involved in inositol phosphate metabolism), tripartite motif containing 39 (*trim39*), sialic acid-binding ig-like lectin 14 (*siglec14*), and killer cell lectin-like receptor b1 (*klrb1*) (involved in the innate immune system) were only differentially expressed 72 h after the salinity change. Of the 308 subpopulation-dependent genes related to salinity change, the highest number of DEGs was observed after salinity decreased (190) and the lowest after salinity increased to 33 ppt. Of these, 74 DEGs (23.94%) were shared between the salinity groups. According to the time course, salinity increased and decreased caused a differential expression of genes involved in the immune response (*aep1*, *nlrc3*, *nlrp3*, *gvinp1*, *L-rhamnose-binding lectins*, *muc2*, and *piscidin*), thyroid metabolism (*dio1* and *notch2*) and ion channel transport (transient receptor potential cation channel subfamily v member 5; *trpv5*) (Figure 8b). Additionally, cytoskeleton and extracellular matrix genes, such as *lox*, *myh3*, *myl10*, desmin (*des*), *col10a1*, and *fn1*, were common between salinities. On the other hand, genes related to the cell cycle, including blm recq-like helicase (*blm*) and baf nuclear assembly factor 1 (*banf1*), response to elevated platelet cytosolic Ca^2+^ (endonuclease domain containing 1; *endod1*), immune-related genes (*cfh*, *gvinp1,* and immunoglobulins), and *plaat4* (involved in glycerophospholipid biosynthesis), were only differentially expressed at 3 ppt. Salinity increased by 10 ppt (to 18 ppt and 28 ppt) caused caspase expression changes (*casp2* and *casp8*), *bcl2l14* related to the apoptosis process, some immune-related genes (*ccl2* and *ccl20*), immunoglobulin lambda constant 1 (*iglc1*), and apolipoprotein L domain-containing protein 1 (*apold1*) (involved in lipid metabolism). The lowest number of genes that showed a difference between the GDA and KIEL groups was detected at a salinity level increased to 33 ppt. Between identified genes were those involved in the notch signaling pathway (*notch2* and *crebbp*), ppar signaling and lipid metabolism (*acsbg2* and *fabp1*), ferroptosis (iron-responsive element-binding protein 2; *ireb2*) and the keratinization process, such as keratin (*krt13*), cornifelin (*cnfn*), and s100 calcium-binding protein a16 (*s100a16*).

## 4. Discussion

Salinity is an important abiotic factor that affects cod activity, distribution, and reproduction in the Baltic Sea. Due to climate change and increased precipitation in northern latitudes, salinity in the Baltic Sea has been expected to decrease [7]. Areas with sea surface salinity less than 7 ppt have expanded since the 1970s, and could cover the whole Baltic Sea in the coming decades [50,51]. The western and eastern Baltic cod populations have already adapted to the marginal salinity of the Baltic Sea [9,52]. The ongoing salinity decline may cause the relocation of cod, e.g., migration of the EBC stock towards the south and west directions. Previous studies have showed the coexistence of WBC and EBC in the Arkona Sea due to differences in depth distribution (WBC occurred mainly in shallower waters <20 m, while EBC were found in deeper waters from 40 to 70 m) [53]. In another scenario, due to genetic differences between stocks, salinity stress may induce selective mortality and local extinction [9].

Previous studies on the adaptation of Atlantic cod to low salinity in the Baltic Sea showed that the mechanisms may be different in divergent subpopulations of cod, which may contribute to a strong and effective reproductive barrier [14,16,34]. However, large-scale research on gene expression has not been conducted. In the present study, the genome-wide oligonucleotide DNA microarray was employed to compare transcriptomic responses to salinity changes in the western and eastern Baltic cod subpopulations represented by fish from the Kiel Bight (KIEL) and the Gulf of Gdańsk (GDA), respectively. The results are in accordance with our previous studies using the RT-qPCR method and transcriptome sequencing [13,14,32]. All individuals used in this study have also been measured in length and weight and showed statistically significant differences between the WBC and EBC subpopulations (Figure 3), which is consistent with previous studies [54]. These results showed a poor condition of the EBC, which may cause difficulties in recovery of the stock [55]. A comparison of the magnitude of expression changes between the GDA and KIEL groups revealed that during salinity reductions, the GDA groups responded more robustly; this may indicate that the EBC subpopulation is better positioned for a low salinity future. Further, we found that these two subpopulations displayed different expression patterns (presumably adaptive responses) to salinity fluctuations. Despite several similarities, 400 DEGs showed statistically significant differences between the KIEL and GDA groups at different time points and salinities. These results provide new insights into the molecular mechanisms underlying salinity adaptation in Baltic cod subpopulations.

The analysis of gene expression revealed many subpopulation-dependent genes involved in immune defense in WBC and EBC, thus indicating that salinity fluctuations have a complex effect on the immune system, which is in accordance with previous studies in teleosts [5,40]. Changes in immune-related pathways, in turn, can affect susceptibility to bacterial, viral, and parasitic infections [56]. Among the subpopulation-dependent genes, there were several transcripts belonging to pattern recognition receptors (PRRs). PRRs are the key sensors of the innate immune system that respond to conserved molecular patterns of microorganisms [57]. Two types of PRRs have been identified in this study, including nucleotide oligomerization domain (NOD)-like receptors (NLRs) and c-type lectin receptors (CLRs). CLRs that include L-rhamnose-binding lectins (*csl2-like* and *sml-like*) showed a subpopulation-dependent pattern during salinity fluctuations throughout the course of time, while fish egg lectins (*fels*) were regulated during salinity elevation. These results suggest that different types of CLR are activated during salinity fluctuations in Baltic cod. In addition to pattern recognition, lectins are involved in inflammation, agglutination, phagocytic reactions, cell proliferation, protein folding, RNA splicing, and molecular trafficking of molecules [58]. Furthermore, transcripts belonging to the NLR family (*nlrp3* and *nlrc3*) showed a subpopulation-dependent pattern under all salinities and times. NLRs play a crucial role in the inflammatory response by forming an inflammasome in the cytosol to protect the organism from pathogens and environmental factors [59]. The results of this study suggest that NLRs may act as sensors to salinity stress, which is in agreement with previous studies in grass carp (*Ctenopharyngodon idella*) [60]. In particular, the expression of CLRs and NLRs in the GDA and KIEL subpopulations depends on the transcript variant, which is in accordance with our previous study, and indicates that alternative splicing supports the response to the variable salinity environment in Atlantic cod [14]. In this study, *cfh*, *cd59*, and *plat* (involved in complement cascade) showed different expression patterns between the EBC and WBC subpopulations. Complement components recognize, opsonize, phagocyte pathogens and promote the inflammatory response [61]. Previous studies have indicated that the complement system response may be salinity specific [62]. In coho salmon (*Oncorhynchus kisutch*) gill tissue, members of this pathway were both down- and up-regulated in response to salinity stress [40]. After 1 h post-salinity change, the *lysozyme g* showed differential expression between the EBC and WBC subpopulations. The lysozyme is a lytic enzyme that lyzes pathogens, and is involved in opsonization, phagocytosis, and activation of the complement cascade [61]. In accordance, previous studies in *Takifugu fasciatus* and common carp (*Cyprinus carpio*) revealed that salinity had an impact on lysozyme activity [63,64]. In another study, *lysozyme g* activity was associated with complement activity in Tilapia (*Oreochromis mossambicus*) after transfer to saltwater [65]. Additionally, the gene identified as *piscidin* belonging to teleost-specific antimicrobial peptides (AMPs) showed a subpopulation-dependent pattern as salinity declined. *Piscidin* is involved in the innate immune response and exhibits antimicrobial activity against various bacteria, viruses, fungi, and parasites [66]. Previous studies have shown that *piscidin* can alter plasma membranes via pore formation [66], and its expression may be affected by acute stress [67]. Together, AMPs, complement components, lectins, and lysozymes create fish mucosal immunity [61]. The mucus layer is a barrier between organisms and the environment, and in addition to its immunological role, it may be involved in osmoregulation [68,69]. Other important constituents of mucus are high molecular weight glycoproteins called mucins. In this study, the expression of several types of mucin differed between the EBC and WBC subpopulations, depending on the course of salinity. *Muc2* was common among all salinities, *I-muc* showed subpopulation-dependent patterns at low salinity, while *muc5ab* and *muc5b* during salinity increased. Further, st3 beta-galactoside alpha-2,3-sialyltransferase 1 (*st3gal1*), involved in mucin type O-glycan biosynthesis, showed different expression patterns between the GDA and KIEL groups. Previous studies on Atlantic salmon (*Salmo salar*) have revealed that the glycosylation process may differ within and between species [70,71]. The expression of mucin transcripts involved in salinity was identified in Arabian pupfish (*Aphanius dispar*) [38], and the salinity-driven modification of mucins has been studied in Atlantic salmon [72]. Mucus cells were activated in the gills of Japanese eel (*Anguilla japonica*) after their transfer to seawater [69]. These results suggest that mucin secretion may be important in adapting to salinity fluctuations in cod from the Baltic Sea and differ between western and eastern subpopulations. Osmotic stress might also affect the innate immune response of fish [73]. In this study, many transcripts annotated as *aep1* were identified during salinity fluctuations, and most of them showed subpopulation-dependent patterns. *Aep1* belongs to the pore-forming proteins (PFPs), widely distributed among teleost genomes, which efficiently kill targets due to the damage to membrane cells by forming a transmembrane pore [74,75]. Studies on aerolysin-like isoforms of zebrafish (*Danio rerio*) showed that they function as innate immune molecules [76]. So far, its involvement in osmoregulation is unknown; however, natterin was also regulated via salinity in Arctic charr (*Salvelinus alpinus*) [77]. Together, these results suggest that salinity fluctuations affect immune-related genes depending on the Baltic cod subpopulation, which affect their immune cascade and, thus, their ability to detect pathogens.

In the present study, salinity stress affected the expression of genes related to programmed cell death (PCD), including the apoptosis, necroptosis, and p53 signaling pathways. Between these genes, several caspases (*casp2*, *casp8*, and *casp13*), proteins from the Bcl2 family (*bax*, *bcl2l14*, and *bnip3*), and members of the tumor necrosis factor receptor superfamily (*tnfrsf9*, *tnfrsf10b*, *tnfrsf14*, and *tnfrsf15*) showed a subpopulation-dependent response. Caspases are highly conserved intracellular cysteine-dependent proteases known for their critical role in mediating apoptosis and inflammatory responses [78]. According to this study, *casp8* previously showed population-dependent regulation in killifish (*Fundulus heteroclitus*) [79]. The members of the Bcl2 family regulate cell apoptosis by inducing or repressing cell death [80]. Different alterations in *bax*/*bcl2* expression ratios can affect mitochondrial cytochrome c release [81], thus inducing cell apoptosis [82]. TNFSFs are inflammatory cytokines that are involved in a variety of pathways ranging from inflammation, lymphocyte maturation, and apoptosis to lymphoid and epithelial tissue development [83]. The influence of salinity on apoptosis has been observed in coho salmon [40] and Arabian pupfish [38]. According to previous studies, programmed cell death is one of the steps in remodeling the gill epithelium in response to salinity fluctuations [38,84].

In this study, genes encoding components of the cytoskeleton and extracellular matrix (ECM) involved in cell structure, motility, intercellular communication, and morphogenesis, such as *col10a1*, *myh3*, *myh7*, *myl3*, *myl10*, *fn1*, *des,* and *lox*, showed differences between the EBC and WBC subpopulations. *Lox* is an enzyme that plays a role in ECM remodeling by cross-linking collagen and elastin [85]. Additionally, genes involved in keratinization (*krt13*, *cnfn*, and *s100a16*) were differentially expressed in the KIEL and GDA groups in response to increased salinity. During the keratinization process, keratins accumulate inside epithelial tissue cells to form a barrier, thereby reducing water loss during dehydration, as seen in Arabian pupfish following salinity [38]. Similar results were obtained in the gills of coho salmon after acclimatization to salinity [40]. Gill epithelium remodeling regulates cell adhesion and signaling [86] and is a well-known component of osmoregulation in fish, studied via transcriptome analysis in rainbow trout (*Oncorhynchus mykiss*) gill ionocytes in freshwater and seawater [87].

Endocrine regulation, including thyroid hormones, is important in salinity acclimatization in euryhaline fish [88]. Studies on the osmoregulatory system in Siberian sturgeon (*Actipenser baeri*) highlighted the importance of the thyroid hormone signaling pathway in this process [36], which has also been confirmed by results from this study. Thyroid hormones regulate carbohydrate, lipid, and steroid metabolism. Additionally, NKA activity and epithelium density can be significantly modified by thyroid hormones during salinity fluctuations [89]. In this study, thyroid hormone signaling was one of the most enriched pathways, and transcripts such as *dio1*, *prkca*, *pik3ca*, *notch2*, and *crebbp* were differentially expressed between the EBC and WBC subpopulations. These results suggest that the impacts of salinity on thyroid hormone homeostasis and the activity of the presented genes depend on the cod subpopulation.

Alterations in the metabolic pathways in response to salinity have been observed in various species [90,91]. The results of this study revealed that salinity fluctuations cause subpopulation-dependent effects on lipid metabolism in Baltic cod. Lipids provide energy to maintain osmotic balance and regulate membrane structure [92,93]. A study on Nile tilapia suggested that fish may use lipids as their main energy source under osmotic stress [37]. Among the DEGs identified, *acsbg2* and *fabp1*, involved in fatty acid metabolism, were differentially regulated during the decrease and increase. These genes were also altered in response to salinity in the delta smelt (*Hypomesus transpacificus*) [94] and in the tongue sole (*Cynoglossus semilaevis*) [95]. Moreover, ceramide synthases (*cers2*, *cers3*, and *cers4*) showed a subpopulation-dependent trend at low and high salinity, depending on the transcript variant. Ceramide synthases reside at the center of sphingolipid metabolism by producing ceramides through de novo synthesis or degradative pathways [96]. Previously, *cers2* was down-regulated in Arctic charr [77] and up-regulated in juvenile Nile tilapia [37]. In this study, the genes *cyp1a1*, *cyp1a2*, and *cyp2d15* that encode cytochrome P450 (CYP) enzymes showed subpopulation-dependent patterns. CYPs are involved in the transformation and metabolism of many endogenous and exogenous compounds in fish, including the biodegradation of xenobiotics. In teleosts, *cyp1a1* has been suggested as an indicator of chemical stress [97], and its important role in acclimatization to higher salinity has been reported in rainbow trout and coho salmon [87,97]. In summary, these results indicate differential metabolic reprogramming in subpopulations of EBC and WBC in response to salinity.

## 5. Conclusions

Gene expression analysis using an oligonucleotide microarray showed the complexity of salinity adaptation in cod from the Baltic Sea, and revealed that eastern and western cod subpopulations respond differently to fluctuations in salinity. Among the subpopulation-dependent genes were those involved in the immune system, which could thus affect susceptibility to pathogens. Moreover, the EBC and WBC subpopulations differ in metabolism reprogramming, gill remodeling, and programmed cell death. This study provides the first general insight into the pathways and functional categories involved in the response of two Baltic cod subpopulations to salinity fluctuations.

## Data Availability

The microarray data were deposited in the National Center for Biotechnology Information’s Gene Expression Omnibus (NCBI GEO) under the accession number GEO: GSE195878.

## Supplementary Materials

The following supporting information can be downloaded at: https://www.mdpi.com/article/10.3390/cells12232760/s1, Figure S1: Venn diagram of DEGs identified in the GDA and KIEL groups; Figure S2: Comparison between experimental groups. (a) Pearson correlation between GDA and KIEL groups; (b) Number of DEGs in GDA experimental groups; (c) Number of DEGs in KIEL experimental groups; Table S1: List of differentially expressed genes in the GDA and KIEL groups; Table S2: DEGs identified in each experimental group. Table S3: List of subpopulation-dependent genes.
