# Supplementary figures and images for: Diverse Transcriptome Responses to Salinity Change in Atlantic Cod Subpopulations
Source: Cells. 2023 Dec 3;12(23):2760. doi: 10.3390/cells12232760 (PMC10706248; doi:10.3390/cells12232760)

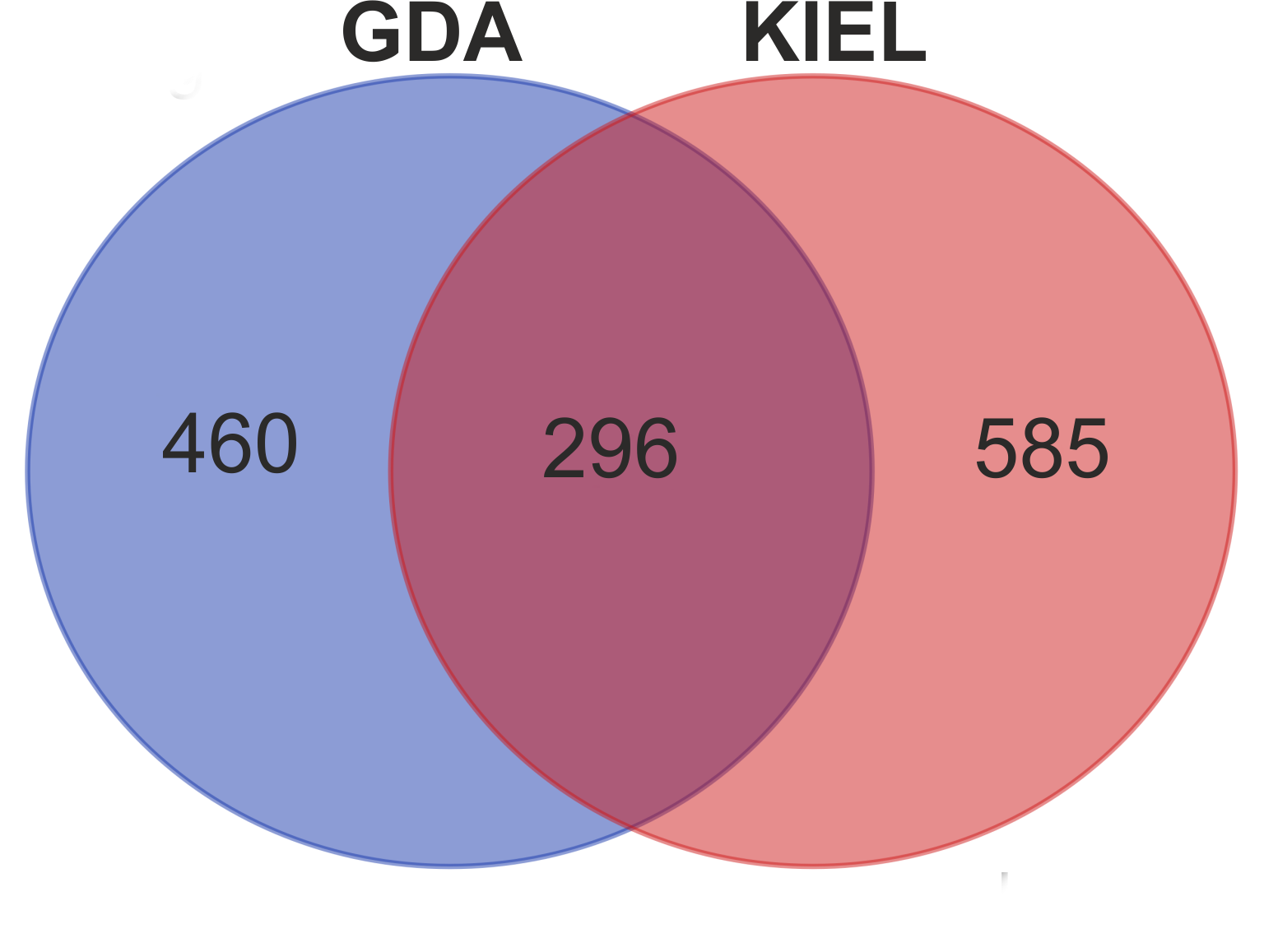

Supplement: Supplementary file 1 [file cells-12-02760-s001.zip › Supplementary Figure S1.png]

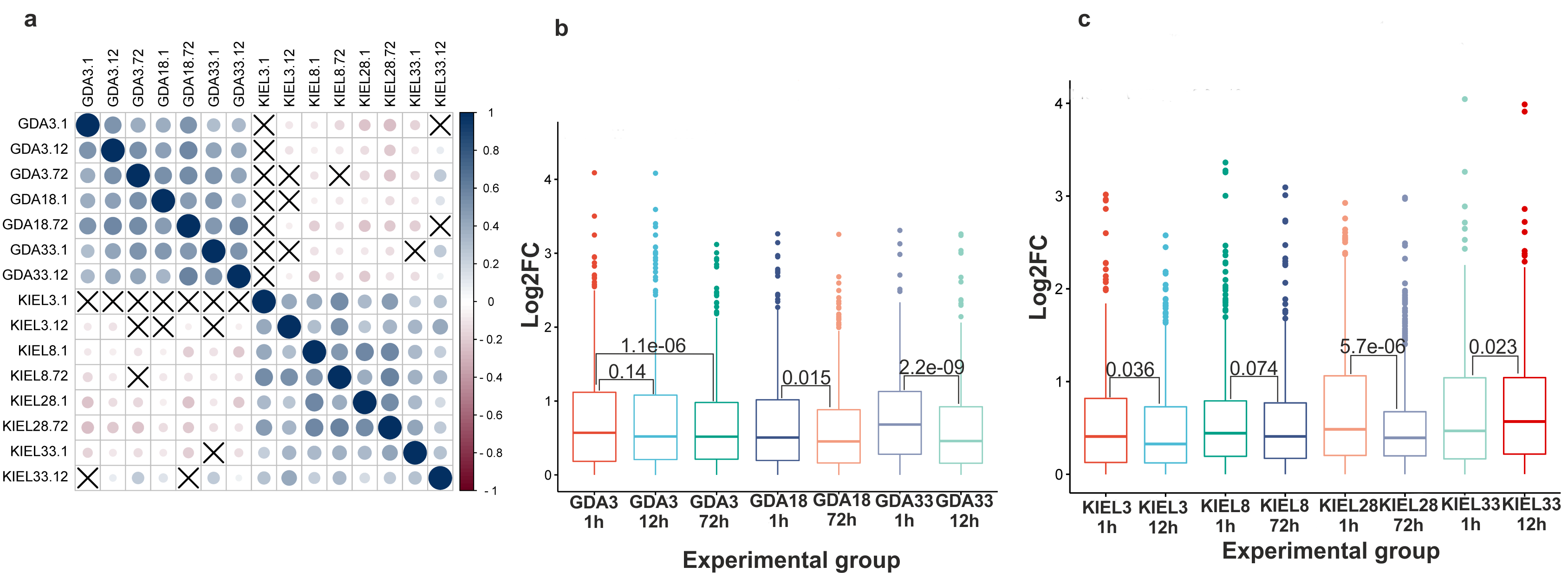

Supplement: Supplementary file 1 [file cells-12-02760-s001.zip › Supplementary Figure S2.png]
